# The British Journals: Animal Chemistry

**Published:** 1839-04

**Authors:** 


### ANIMAL CHEMISTRY.

### *Detection of Urea in the Blood of a patient who died of Cholera.* By Harry Rainy, m.d., Glasgow.

[This is an important communication, and highly creditable to the author. At the time of his analysis, Dr. R. was not aware of there existing any distinct account of urea being found in human blood, or of its precise quantity being determined; he has since found that Dr. Christian had obtained satisfactory evidence of its presence in dropsy. We quote only the chemical details of this case.]

During the whole progress of the disease the urinary secretion was nearly arrested, the whole quantity secreted during eleven days being only 36 ounces, including a small quantity found in the bladder after death. This gives an average of little more than 3 ounces in twenty-four hours. The urine coagulated by heat

After separating the albuminous matter, urea was detected in it by nitric acid, but the proportion was much smaller than in healthy urine. From this circumstance I thought it probable that urea had accumulated in the blood.

In order to decide this point, 4 ounce measures of blood were taken from the larger vessels and heart; it was partly fluid, partly in small coagula. As the serum could not be separated in sufficient quantity for examination, the whole was mixed with 12 ounce measures of alcohol, well stirred for some minutes, and then allowed to digest for a day at a moderate temperature. The albuminous and colouring matter was precipitated in reddish brown flakes. The alcoholic liquor partly floated on the top. The mixture being thrown on a filter of fine cotton cloth, 10 ounce measures of fluid passed through, by the aid of gentle compression, the rest being retained by the spongy precipitate. This filtered fluid was transparent and almost colourless. It was evaporated at a temperature not exceeding 160° Fah. During this process it became turbid, and deposited a considerable quantity of fluid oily matter, which was separated. When reduced to the consistence of a thin syrup it had a decidedly urinous smell; and a minute portion being tested, yielded distinct pearly scales with nitric acid and with oxalic acid. The syrupy fluid was still turbid, apparently from the presence of oily matter, probably phosphoric fat. In order to separate this matter the extract was diluted with a little water, to render it perfectly fluid. It was then agitated with a small portion of ether, which dissolved the oily matter, and left the watery fluid colourless and almost transparent. This fluid was again evaporated, at a very gentle heat, to the consistence of a thin syrup; and nitric acid being added, there was a slight effervescence, followed by a deposition of pearly crystalline scales. These being compressed between folds of filtering paper and dried, weighed $5\frac{1}{10}$ grains. They had all the characters of the nitrate of urea, and, according to Prout's analysis, may be considered equivalent to $2\frac{7}{10}$ grains of urea.

This quantity, then, was actually separated from 4 ounce measures of blood; but as the whole mixture of blood and alcohol measured 16 ounces, and the filtered liquor measured only 10 ounces, it is evident that the filtered liquor contained only $\frac{10}{16}$th of the urea present in the blood. From these data it will follow that the whole urea actually present in the blood amounted to $2\frac{7}{10} \times \frac{16}{10} = 4\frac{3}{10}$ grains, or rather more than one grain to each ounce measure of blood, without making any allowance for the small quantity remaining in the fluid, to which the nitric acid was added.

*London Medical Gazette. Jan. 5, 1839.*

